# Sedation practices in patients intubated in the emergency department compared with those in patients in the intensive care unit

**DOI:** 10.62675/2965-2774.20250247

**Published:** 2025-05-07

**Authors:** Jariya Sereeyotin, Christopher Yarnell, Sangeeta Mehta

**Affiliations:** 1 Chulalongkorn University King Chulalongkorn Memorial Hospital and Faculty of Medicine Department of Anesthesiology Bangkok Thailand Department of Anesthesiology, Division of Critical Care Medicine, King Chulalongkorn Memorial Hospital and Faculty of Medicine, Chulalongkorn University - Bangkok, Thailand.; 2 University of Toronto Interdepartmental Division of Critical Care Medicine Department of Medicine Toronto Ontario Canada Department of Medicine, Sinai Health, Interdepartmental Division of Critical Care Medicine, University of Toronto - Toronto, Ontario, Canada.; 3 Scarborough Health Network Research Institute Toronto Ontario Canada Scarborough Health Network Research Institute - Toronto, Ontario, Canada.

**Keywords:** Sedation, Intubation, Respiration, artificial, Hypnotics and sedatives, Propofol, Ketamine, Fentanyl, Benzodiazepines, Critical Illness, Emergency service, hospital, Intensive care units

## Abstract

**Objective:**

This study aimed to compare sedation management during and after intubation in the emergency department with that in the intensive care unit.

**Methods:**

This was a single-center retrospective cohort study of adults who were intubated in the emergency department or intensive care unit and who received mechanical ventilation between January 2018 and February 2022. We collected data from electronic medical records. The primary outcome was the duration from intubation to the first documentation of light sedation, which was defined as a Sedation Agitation Scale score of 3 - 4.

**Results:**

This study included 264 patients, 95 (36%) of whom were intubated in the emergency department and 169 (64%) in the intensive care unit. With respect to the anesthetic agents used for intubation, ketamine was the most frequently used drug in the emergency department and was used more frequently than in the intensive care unit (61% *versus* 40%; p = 0.001). Propofol was the predominant sedative used in the intensive care unit, with a higher prevalence than in the emergency department (50% *versus* 33%; p = 0.01). Additionally, benzodiazepines and fentanyl were more frequently used in the intensive care unit (39% *versus* 6%; p < 0.001 and 68% *versus* 9.5%; p < 0.001, respectively). Within 24 hours after intubation, 68% (65/95) of the emergency department patients and 82% (138/169) of the patients intubated in the intensive care unit achieved light sedation, with median durations of 13.5 hours and 10.5 hours, respectively. Patients who were intubated in the emergency department were less likely to achieve light sedation at 24 hours (adjusted hazard ratio 0.64; p = 0.04; 95%CI, 0.42 - 0.97).

**Conclusion:**

Compared with intensive care unit patients, critically ill patients who were intubated in the emergency department are at risk of deeper sedation and a longer time to achieve light sedation.

## INTRODUCTION

Effective sedation management is crucial for facilitating intubation by ensuring comfort and promoting patient—ventilator synchrony in mechanically ventilated adults. However, this management method poses challenges in both the emergency department (ED) and the intensive care unit (ICU) due to limited physiological reserves and the potential for serious complications in critically ill patients.^(1,2)^ Moreover, inappropriate or excessive sedation has been linked to adverse outcomes in these patients.^(3-5)^

Several studies have reported that deep sedation in mechanically ventilated patients during the first 48 hours after ICU admission is associated with increased risks of death, delirium and delayed extubation.^(6-10)^ Despite the 2018 Pain, Agitation/Sedation, Delirium, Immobility and Sleep Disruption (PADIS) guidelines,^(5)^ which recommend a stepwise approach for pain management and light sedation in critically ill mechanically ventilated adults, deep sedation is commonly used for intubated patients in the ED. In a recent multicenter, prospective cohort study of 324 patients receiving mechanical ventilation (MV) in the ED, 52.8% were deeply sedated [defined as a Sedation Agitation Scale (SAS) score of 1 or 2], which continued throughout the first 48 hours of ICU admission.^(11)^ Therefore, early emphasis on analgosedation with sedation minimization in critically ill patients may be beneficial.

Many critically ill patients are initially intubated in the ED prior to transfer to the ICU, yet few studies have evaluated sedation practices for these patients.^(11,12)^ The overall purpose of our study was to compare sedation management during and after intubation in patients intubated in the ED compared with those intubated in the ICU. Because our study period included the COVID-19 pandemic, during which rapid sequence intubation (RSI) was recommended for suspected or confirmed COVID-19 patients,^(13,14)^ we also explored changes in sedation management prior to and during the COVID-19 pandemic.

## METHODS

This retrospective cohort study was conducted at a tertiary care university-affiliated hospital and was approved by the Sinai Health Research Ethics Board (REB#23-0002-C) on February 15^th^, 2023. Owing to the retrospective research design and the fact that no identifying information would be collected, the need for informed consent was waived. Trained reviewers screened and identified eligible consecutive patients from an ICU research screening database and electronic medical records prior to (January 1^st^, 2018-January 31^st^, 2020) and during the COVID-19 pandemic (February 1^st^, 2020-February 28^th^, 2022). We utilized published methodology for chart review studies.^(15)^

The study included adult patients aged 18 years or older who were intubated either in the ED or within the first 24 hours of ICU admission and who later received MV. The exclusion criteria were as follows: need for deep sedation or neuromuscular blocking agents after intubation (e.g., therapeutic hypothermia after cardiac arrest, moderate to severe acute respiratory distress syndrome, or status epilepticus); death within 48 hours of intubation; transfer to another department after intubation; and not expected to achieve light sedation within 48 hours (e.g., intracranial hemorrhage, brainstem infarction, or encephalopathy).

In this study, there was no local protocol available for the sedation management of mechanically ventilated patients in the ED or ICU. During intubation, sedation decisions are made primarily by the intubating physician and are individualized based on the patient's clinical presentation.

The primary outcome was the time from intubation to the first documentation of light sedation, which was assessed within the first 24 hours and defined as an SAS score of 3 - 4. The secondary outcomes included the SAS score at 6, 12, 24 and 48 hours after intubation; the duration of MV; hospital length of stay; 28-day mortality; and discharge disposition.

Secondary analyses were conducted to evaluate the time from intubation to the first documentation of light sedation. These analyses were performed for the periods prior to and during the pandemic and included subgroup analysis of patients with a baseline Glasgow Coma Scale (GCS) score > 8 (ranging from 9 - 15).

The data collected included demographic information and clinical variables: the baseline GCS score, Acute Physiologic Assessment and Chronic Health Evaluation (APACHE) II score, COVID-19 infection status, underlying conditions, and reasons for intubation. Additionally, the intubation technique (non-RSI or RSI, which is defined as the administration of sedative agents followed by rapid onset neuromuscular blocking agents without positive pressure ventilation unless necessary), and the specialty of the individual performing the intubation were recorded. The types and dosages of drugs used for intubation and during the postintubation period were also documented.

### Statistical analysis

Demographic variables are presented as descriptive statistics, and categorical data are presented as frequencies (percentages). For continuous data, the Kolmogorov—Smirnov test was used to assess normality, and the data are expressed as the means (standard deviation [SD]) or median (interquartile range [IQR]) as appropriate. The primary outcome was analyzed using a Cox proportional hazards model, adjusting for covariates potentially relevant to delays in achieving light sedation. The covariates included age, APACHE II score, obesity (body mass index ≥ 30kg/m^2^), renal insufficiency (GFR < 30mL/min/1.73m^2^ or receiving dialysis), end-stage liver disease, baseline GCS score, and the administration of benzodiazepines and neuromuscular blocking agents after intubation. For the primary outcome, we report the hazard ratio of achieving light sedation according to patient location at the time of intubation (ED *versus* ICU).

The secondary analyses for the periods prior to and during the pandemic, as well as subgroup analyses focusing on patients with a baseline GCS > 8, were conducted using the same statistical methods as were used to assess the primary outcome.

All analyses were performed using SPSS software version 28, and a two-sided p value < 0.05 was considered statistically significant.

## RESULTS

**Figure 1:**
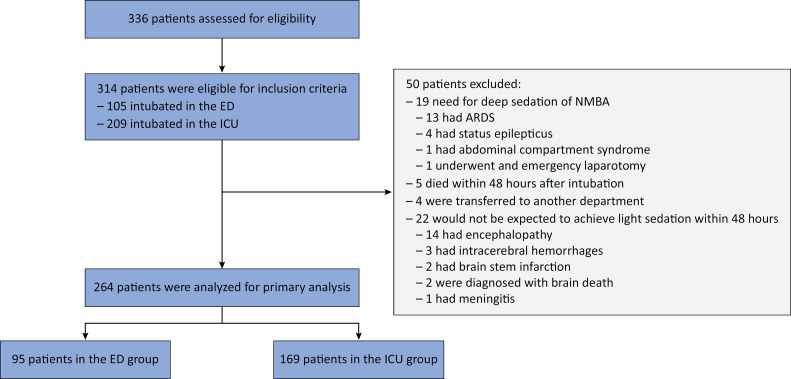
Flow chart of participants in a study of sedation practices in patients intubated in the emergency department compared with those in patients in the intensive care unit.

Between January 2018 and February 2022, 314 patients were eligible for the inclusion criteria, of whom 50 patients were excluded (Figure 1). Ultimately, 264 patients were included in the primary analysis, with 95 (36%) intubated in the ED and 169 (64%) intubated in the ICU. The mean (SD) age was 60 (18) years, and 153 (58%) were male. The two groups were similar at baseline prior to intubation, with the exception that a greater proportion of patients in the ED group had a GCS ≤ 8 (68% *versus* 25%) and the patients in the ICU group had a greater mean age (63 *versus* 56 years). The most common reason for intubation in the ED was neurological dysfunction, whereas respiratory failure was the most common reason for intubation in the ICU. A majority of the patients with a GCS score ≤ 8 had metabolic causes (43%), followed by simple seizures (28%), drug overdose (20%), postcardiac arrest (6.3%), and other causes (2%). Patient characteristics are shown in table 1.

ED - emergency department; ICU - intensive care unit; NMBA - neuromuscular blocking agent; ARDS – acute respiratory distress syndrome.

**Table 1:** Patient characteristics prior to endotracheal intubation

Variables		Emergency department n = 95	Intensive care unit n = 169
Age (years)		56 (21)	63 (15)
Male sex		55 (58)	98 (58)
BMI (kg/m^2^)		26 (21 - 28)	26 (23 - 31)
	Race*		
	White	68 (72)	110 (65)
	Black	9 (9)	10 (6)
	Other person of color	18 (19)	49 (29)
APACHE II		22 (17 - 27)	22 (17 - 27)
eGFR (mL/min/1.73m^2^)		100 (58 - 138)	85 (51 - 128)
Baseline GCS before intubation			
	GCS ≤ 8	61 (68)	36 (25)
	GCS > 8	29 (32)	110 (75)
COVID infection			
	Positive	6 (6)	6 (4)
	Negative	89 (94)	163 (96)
Underlying disease			
	Hypertension	34 (36)	72 (43)
	Diabetes mellitus	26 (27)	40 (24)
	COPD	9 (9)	20 (12)
	Renal insufficiency	12 (13)	28 (17)
	Hepatic dysfunction	10 (11)	27 (16)
	End-stage liver disease	0 (0)	3 (2)
Reason for intubation			
	Airway obstruction	8 (8)	11 (7)
	Respiratory failure	25 (26)	107 (63)
	Cardiogenic shock	4 (4)	4 (2)
	Neurological dysfunction	46 (48)	22 (13)
	Sepsis	3 (3)	24 (14)
	Cardiac arrest	7 (7)	4 (2)
	Other	2 (2)	0 (0)

1 BMI - body mass index; APACHE II - Acute Physiologic Assessment and Chronic Health Evaluation II score; eGFR - estimated glomerular filtration rate; GCS - Glasgow Coma Scale; COPD - chronic obstructive pulmonary disease.

* Race was visually identified and recorded in the ICU research database by the research coordinator. The results are presented as the means (standard deviations), n (%) or medians (interquartile ranges).

### Primary outcome and sedation agitation scale

**Figure 2:**
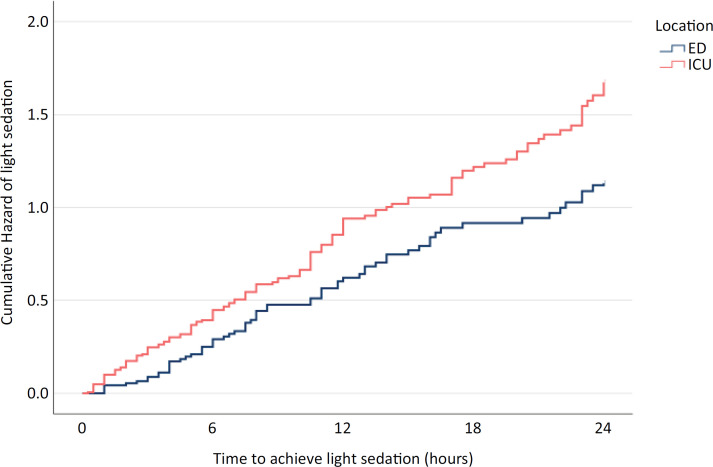
Cumulative hazard of the time to achieve light sedation at 24 hours stratified by the patient's location of intubation.

Within the first 24 hours after intubation, 68.4% (65/95) of the ED patients and 81.7% (138/169) of the ICU patients achieved light sedation, with median durations of 13.5 (6 - 24) hours and 10.5 (4 - 21.3) hours, respectively. Patients who were intubated in the ED were less likely to achieve light sedation at 24 hours, with an adjusted hazard ratio of 0.64 (p = 0.04; 95%CI, 0.42 - 0.97) (Table 1S - Supplementary Material). Cumulative hazard curves assessing the time from intubation to the first documentation of achieving light sedation are shown in figure 2. However, when the data were analyzed separately before and during the COVID-19 pandemic, no significant associations were detected between patient location at intubation and the probability of achieving light sedation within 24 hours (Tables 2S and 3S - Supplementary Material).

Within the first 24 hours after intubation, the median time to achieve light sedation was 13.5 hours for patients intubated in the emergency department and 10.5 hours for those intubated in the intensive care unit. The mean hazard ratio for achieving light sedation at 24 hours was 0.69 for the emergency department (p = 0.01; 95% confidence interval 0.51 - 0.92).

ED - emergency department; ICU - intensive care unit.

A subgroup analysis of 139 patients with baseline GCS scores > 8 showed similar findings to that in the primary analysis: patients intubated in the ED were less likely to achieve light sedation within 24 hours (adjusted hazard ratio 0.603; p = 0.04; 95%CI, 0.37 - 0.98) (Figure 1S - Supplementary Material). The median time to achieve light sedation was 14 (6 - 22) hours for ED patients and 7.5 (4 - 11) hours for ICU patients. However, after multivariate adjustment, the association between intubation location and the probability of achieving light sedation was no longer significant (Table 4S - Supplementary Material).

**Figure 3:**
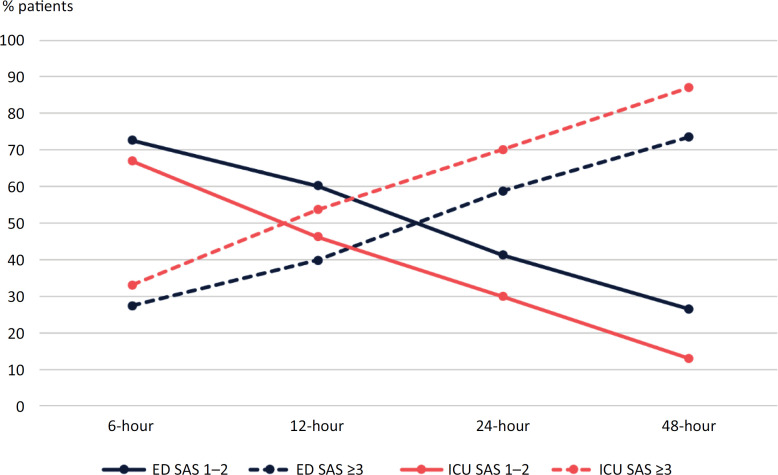
Sedation Agitation Scale at each time point in the emergency department and intensive care unit patients.

Deep sedation (SAS 1 or 2) was more frequently observed in the ED group at all time points, especially at 12 and 48 hours (60.2% *versus* 46.3%; p = 0.03; and 26.5% *versus* 13.0%; p = 0.02, respectively) (Figure 3). Details regarding the number of patients who were deeply or lightly sedated at each timepoint are provided in table 5S (Supplementary Material).

The Sedation Agitation Scale (SAS) at each time point is shown for patients intubated in the emergency department and the intensive care unit. Deep sedation (SAS 1 or 2) was more frequently observed in the emergency department group, and a higher prevalence of light sedation (SAS 3 or 4) was found in the intensive care unit group at all time points, with statistically significant differences at 12 and 48 hours (60.2% *versus* 46.3%; p = 0.03; and 26.5% *versus* 13.0%; p = 0.02, respectively, for deep sedation; 39.8% *versus* 53.7%; p = 0.03 and 73.5% *versus* 87.0%; p = 0.02, respectively, for light sedation).

ED - emergency department; ICU - intensive care unit; SAS - Sedation Agitation Scale.

### Intubation technique, drug types and drug dosages

Rapid sequence intubation was significantly more common in the ED than in the ICU (82.1% *versus* 30.2%; p < 0.001). During the COVID-19 pandemic, the incidence rate of RSI significantly increased from 75% to 92.3% (p = 0.03) in the ED and from 17.3% to 50.8% in the ICU (p < 0.001). The intubation techniques used prior to and during the COVID-19 pandemic in the ED and ICU are presented in table 6S and figure 2S (Supplementary Material).

With respect to the anesthetic agents used for intubation (Table 2), ketamine was the most commonly used sedative in the ED and was used more frequently than in the ICU (61% *versus* 40% patients; p = 0.001). Propofol was the predominant sedative used in the ICU, with a higher prevalence than in the ED (50% *versus* 33%; p = 0.01). Additionally, benzodiazepines, opiates and topical xylocaine were more frequently used in the ICU (39% *versus* 6%; p < 0.001; 68% *versus* 10%; p < 0.001; and 15% *versus* 3%; p = 0.003; respectively).

**Table 2:** Details of the intubator specialty and sedative, analgesic, and paralytic medications used for intubation and postintubation in the emergency department *versus* the intensive care unit

Intubation details			Emergency department n = 95	Intensive care unit n = 169	p value
Intubator specialty					
		ED physician	79 (83.2)	0	< 0.001
		Critical care physician	8 (8.4)	169 (100)	< 0.001
		Anesthesiologist	8 (8.4)	0	< 0.001
Induction drug administration					
		Xylocaine topical*	3 (3.2)	25 (14.8)	0.003
		Fentanyl	9 (9.5)	115 (68)	< 0.001
		Dosage (mcg/kg)	1.1 (0.6 - 1.4)	1.0 (0.6 - 1.4)	0.50
		Ketamine	58 (61.1)	68 (40.2)	0.001
		Dosage (mg/kg)	1.2 (0.8 - 1.4)	0.9 (0.5 - 1.2)	< 0.001
		Propofol	31 (32.6)	85 (50.3)	0.01
		Dosage (mg/kg)	1.2 (0.9 - 1.7)	0.7 (0.4 - 1.0)	< 0.001
		Midazolam	6 (6.3)	66 (39.1)	< 0.001
		Dosage (mg/kg)	0.07 (0.03 - 0.1)	0.02 (0.02 - 0.03)	0.02
		Etomidate	1 (0.01)	0	< 0.001
		Dosage (mg/kg)	0.43	0	< 0.001
		Rocuronium	44 (46.3)	64 (37.9)	0.18
		Dosage (mg/kg)	1.2 (1.0 - 1.4)	0.8 (0.7 - 1.1)	< 0.001
		Succinylcholine	36 (37.9)	0	< 0.001
		Dosage (mg/kg)	1.4 (1.2 - 1.9)	0	< 0.001
Postintubation drug administration					
		Opioids	24 (25.3)	116 (68.6)	< 0.001
		Opioid dosage (fentanyl equivalent†)			
		Bolus (mcg/kg)	0.9 (0.3 - 1.3)	0.5 (0.3 - 1.2)	0.37
		Infusion (mcg/kg/hour)	0.8 (0.6 - 1.5)	0.9 (0.6 - 1.3)	0.76
		Ketamine	16 (16.8)	8 (4.7)	0.001
		Ketamine dosage			
		Bolus (mg/kg)	0.2 (0.1 - 0.4)	0.7 (0.5 - 1.2)	0.09
		Infusion (mg/kg/hour)	0.4 (0.3 - 0.5)	0.06 (0.05 - 0.15)	0.07
		Propofol	48 (50.5)	110 (65.1)	0.02
		Propofol dosage			
		Bolus (mg/kg)	0.6 (0.3 - 1.1)	0.7 (0.4 - 0.8)	0.78
		Infusion (mcg/kg/min)	30 (20 - 50)	30 (20 - 40)	0.046
		Benzodiazepines	32 (33.7)	14 (8.3)	< 0.001
		Benzodiazepine dosage (midazolam equivalents‡)			
		Bolus (mg/kg)	0.04 (0.03 - 0.09)	0.04 (0.02 - 0.05)	0.37
		Infusion (mg/kg/hour)	0.05 (0.03 - 0.08)	0.04 (0.03 - 0.05)	0.44
		Intermittent NMBA administration	4 (4.2)	9 (5.3)	0.69
		Method of opioid administration, peri-intubation			
		Bolus	12 (12.6)	43 (25.4)	
		Infusion	11 (11.6)	30 (17.8)	< 0.001
		Both	3 (3.2)	77 (45.6)	
		None	69 (72.6)	19 (11.2)	
		Method of sedative administration, peri-intubation			
		Bolus	12 (12.6)	35 (20.7)	
		Infusion	3 (3.2)	9 (5.3)	0.15
		Both	78 (82.1)	117 (69.2)	
		None	2 (2.1)	8 (4.7)	

ED - emergency department; NMBA - neuromuscular blocking agent. The most common benzodiazepine used in the emergency department and intensive care unit was midazolam.

* Xylocaine topical was used for standard intubation (not for the awake fiberoptic technique);

† fentanyl equivalent parenteral dose; fentanyl 0.1mg = morphine 10mg = hydromorphone 1.5mg (Evidences: Barr J, Fraser GL, Puntillo K, Ely EW, Gélinas C, Dasta JF, etc.; American College of Critical Care Medicine. Clinical practice guidelines for the management of pain, agitation, and delirium in adult patients in the intensive care unit. Crit Care Med. 2013:41(1);263-306);

‡ Midazolam equivalent parenteral dose; midazolam 1.5mg = diazepam 5mg (Evidences: Reuben Straye. www.maimonidesem.org/blog/benzodiazepines). Data are shown as n (%) or median (interquartile range).

Higher doses of sedatives and neuromuscular blocking agents were used for intubation in the ED, whereas no significant difference in opioid dosage was found. We identified 3 patients intubated in the ED who received neuromuscular blocking agents alone without documented sedatives: 2 were diagnosed with drug intoxication and had a baseline GCS score of 3 with no improvement after naloxone administration, whereas the other had a baseline GCS score of 5 and was intubated due to COVID-19 infection and septic shock.

After intubation, opioids were less frequently used (25.3% *versus* 68.6%; p < 0.001), whereas ketamine and benzodiazepines were more frequently used (16.8% *versus* 4.7%; p = 0.001; and 33.7% *versus* 8.3%; p < 0.001, respectively) in the ED than in the ICU. The bolus and infusion dosages were similar in the ED and ICU, with the exception of higher propofol infusion rates in the ED [30 (20 - 50) *versus* 30 (20 - 40) mcg/kg/min; p = 0.046].

Compared with that used prior to the COVID-19 pandemic, rocuronium for intubation was used at higher doses both in the ED and the ICU during the COVID-19 pandemic [0.9 (0.6 - 1.0) *versus* 1.3 (1.2 - 1.6) mg/kg; p < 0.001 in the ED; and 0.7 (0.6 - 0.8) *versus* 1.1 (0.7 - 1.4) mg/kg; p < 0.001 in the ICU]. After intubation, there was a higher prevalence of propofol and benzodiazepine use, as well as higher doses of propofol infusion in the ICU, whereas a higher prevalence of ketamine was observed in the ED (Tables 7.1S and 7.2S - Supplementary Material).

Following ICU intubation, patients had SAS scores recorded every 4 hours. In the ED, the GCS score was documented postintubation, but no sedation assessment tool was used until the patient arrived in the ICU, at which point the SAS score was recorded every 4 hours.

### Other outcomes

**Figure 4:**
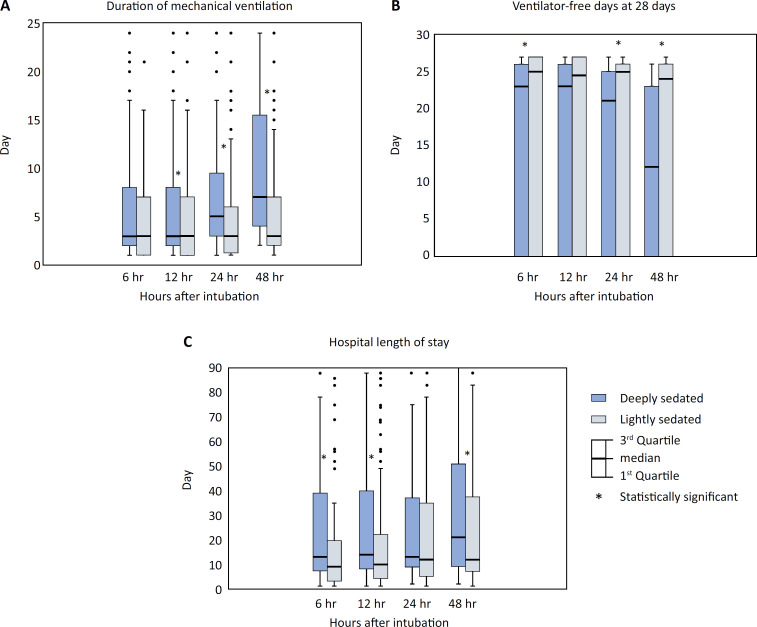
Duration of mechanical ventilation, ventilator-free days at 28 days, and length of hospital stay between the deeply sedated and lightly sedated groups.

Compared with the ED group, the ICU group had a longer duration of MV and extended hospital stay and higher 28-day mortality rates (4 (2 - 9) *versus* 2 (1 - 3) days; p < 0.001; 14 (8 - 47) *versus* 8 (3 - 16) days; p < 0.001 and 37.7% *versus* 12.3%; p < 0.001, respectively) (Table 3). However, patients who were deeply sedated at any time point had longer MV days and hospital stays and fewer ventilator-free days at 28 days than did those in the lightly sedated group (Figure 4). In addition, deeply sedated patients were less likely to be discharged home and more likely to be transferred to a rehabilitation facility (Table 8S - Supplementary Material).

**Table 3:** Intensive care unit outcomes between the emergency department and intensive care unit groups

Outcomes		Emergency department n = 95	Intensive care unit n = 169	p value
Duration of mechanical ventilation (days)		2 (1 - 3)	4 (2 - 9)	< 0.001
Ventilator-free days at 28 days		26 (24 - 27)	17 (0 - 25)	< 0.001
Hospital length of stay (days)		8 (3 - 16)	14 (8 - 47)	< 0.001
28-day mortality		10/81 (12.3)	61/162 (37.7)	< 0.001
Discharge disposition				< 0.001
	Home	52 (54.7)	30 (17.8)	
	Death	10 (10.5)	77 (45.6)	
	Rehabilitation or long-term care institution	21 (22.1)	23 (13.6)	
	Acute care hospital	12 (12.6)	39 (23.1)	

Data are shown as the median (interquartile range), N/total collected data (%) or n (%).

(A) The duration of mechanical ventilation was significantly longer in the deeply sedated group than in the lightly sedated group at any time point at 12, 24 and 48 hours after intubation. (B) Patients who were lightly sedated at 6, 24 and 48 hours after intubation had more ventilator-free days at 28 days than did those who were deeply sedated. (C) Patients who were deeply sedated at 6, 12 and 24 hours after intubation had significantly longer hospital stays than lightly sedated patients did.

Deeply sedated is defined as a Sedation Agitation Scale score of 1 - 2; lightly sedated is defined as a Sedation Agitation Scale score of ≥ 3. * Statistically significant.

## DISCUSSION

In this single-center retrospective cohort study, patients who were intubated and had initiated invasive ventilation in the ED took longer to achieve light sedation compared with patients who were intubated in the ICU. Moreover, deep sedation was more frequently observed in the ED group during the first 48 hours of MV.

Our study underscores the impact of ED sedation practices on the outcomes of mechanically ventilated patients, including fewer ventilator-, ICU- and hospital-free days.^(11,12)^ Our findings align with those of the ED-SED study, which revealed a greater frequency of deep sedation in patients who were intubated in the ED for up to 48 hours than in those who were intubated in the ICU.^(11)^

We observed a greater incidence of RSI in the ED than in the ICU, which may be attributed to the training of ED physicians and different patient profiles. In the ED, intubations were mostly performed due to neurological dysfunction, whereas in the ICU, they were mainly because of respiratory failure and sepsis. As a result, ICU patients are at greater risk of serious complications, such as life-threatening hypoxemia and hemodynamic collapse,^(16)^ which reduces their tolerance for the RSI. In these cases, RSI alternatives are recommended.^(17,18)^

There is limited high-quality evidence on the optimal sedative doses for the intubation of critically ill adults.^(17,19,20)^ Higher doses of sedatives and neuromuscular blocking agents are used in the ED, even though neurologic dysfunction is the most common reason for intubation. Three patients with low baseline GCS scores (3 - 5) were intubated in the ED using neuromuscular blocking agents alone without sedation, highlighting an opportunity for improvement.

In a study of intubation practices in 3,659 critically ill patients across 29 countries, the main reasons for intubation were respiratory failure (52%) and neurological dysfunction (31%), which is similar to the findings in the current study. In their study, the most commonly used drugs for induction were propofol (41%), midazolam (36%), etomidate (18%) and ketamine (14%).^(17)^ In contrast, in our study ketamine was the most commonly used drug in the ED (61%), whereas propofol and ketamine were the most frequently used drugs in the ICU (50% and 40%, respectively).

With respect to postintubation analgesia, only 25.3% of patients in the ED received opioids. Similarly, the ED-SED study reported that 28.4% of 324 patients did not receive analgesia, and 10.8% received neither analgesia nor sedation.^(11)^ This practice is inconsistent with the 2018 PADIS Guidelines,^(5)^ which recommend prioritizing analgesia first via a stepwise approach for pain and sedation management in critically ill adults.

Benzodiazepines, although unsuitable for long-term sedation and no longer recommended for critically ill patients,^(21,22)^ remain indicated for specific conditions, such as seizures and withdrawal syndromes. In our study, 33.7% of the ED patients received benzodiazepines compared to 8.3% of the patients in the ICU. Among the ED patients who received benzodiazepines postintubation, 5 were diagnosed with seizure/status epilepticus, and 3 had suspected alcohol withdrawal. Nevertheless, even when these patients were excluded, benzodiazepine use remained higher, and the time to achieve light sedation was longer in the ED than in the ICU. Thus, the inappropriate use of sedatives, as well as the lack of standardized sedation assessment tools^(23)^ in the ED, likely contributed to the delayed achievement of light sedation at 24 hours.

Differences in sedation practices between the ED and ICU are also influenced by the boarding of critically ill patients in the ED, where staffing constraints may hinder the implementation of light sedation and sedation assessment tools.^(24-26)^ In our study, patients who were intubated in the ED typically stayed there only briefly before being transferred to the ICU.

To our knowledge, this is the first study comparing intubation and postintubation sedation practices between the ED and ICU. The strengths of the present study include detailed data on medications and dosages, evaluations prior to and during the COVID-19 pandemic, complete patient follow-up, and identification of predictors of deep sedation. Our study has several limitations. First, it was conducted at a single center. Despite this, the characteristics of our study population and the high prevalence of deep sedation in the ED align closely with those of previous studies. Second, the primary outcome—time from intubation to first documentation of light sedation—depends on the frequency of the nurse's assessment and may not reflect the actual time to light sedation. To address this limitation, we collected sedation scores at four specific time points. The data were collected in the same direction: a greater frequency of deep sedation at every time point and a longer time required to achieve light sedation were found in the ED group. Third, we lack data on potential confounders, such as the intubator's specialty and experience, between the location of intubation and postintubation sedation use. Finally, we were unable to evaluate the associations between deep sedation and longer-term outcomes (e.g., 90-day mortality and cognitive outcomes).

## CONCLUSION

Patients who were intubated in the emergency department were more deeply sedated and took longer to achieve light sedation than patients who were intubated in the intensive care unit.
