# Supplementary material for: Sedation practices in patients intubated in the emergency department compared with those in patients in the intensive care unit
Source: Crit Care Sci. 2025 May 7;37:e20250247. doi: 10.62675/2965-2774.20250247 (PMC12094695; doi:10.62675/2965-2774.20250247)
Supplement: Supplementary file 1 [file 2965-2774-ccsci-37-e20250247-Suppl01.pdf]

Critical Care Science
https://doi.org/10.62675/2965-2774.20250247

Sedation practices in patients intubated in the emergency department compared with those in patients in the intensive care unit

Jariya Sereeyotin[1,2], Christopher Yarnell[2,3], Sangeeta Mehta[2]

Table 1S - Multivariable cox regression analysis for patient location as a predictor for achieving light sedation at 24 hours after adjusting for potential covariates

Variables	Hazard ratio	95% confidence interval	p value
Location of intubation (ED)	0.64	0.42 - 0.97	0.04
Age (1-year increment)	1.00	0.99 - 1.01	0.93
Obesity (BMI ≥ 30kg/m^2)	1.07	0.74 - 1.55	0.72
APACHE II (1-point increments)	0.98	0.96 - 1.01	0.23
Baseline GCS ≤ 8	0.78	0.53 - 1.15	0.21
Renal insufficiency	0.72	0.44 - 1.20	0.21
End-stage liver disease	0.41	0.54 - 3.02	0.38
Postintubation benzodiazepines administration	1.25	0.78 - 1.99	0.36
Postintubation intermittent NMBA administration	0.68	0.30 - 1.57	0.37

ED - emergency department; BMI - body mass index; APACHE II - Acute Physiologic Assessment and Chronic Health Evaluation II; GCS - Glasgow Coma Scale; NMBA - neuromuscular blocking agents.

Table 2S - Multivariable cox regression analysis for patient location as a predictor for achieving light sedation at 24 hours; prior to the COVID-19 pandemic

Variables	Hazard ratio	95% confidence interval	p value
Location of intubation (ED)	0.76	0.41 - 1.41	0.39
Age (1-year increment)	1.00	0.99 - 1.01	0.75
Obesity (BMI ≥ 30kg/m^2)	0.89	0.55 - 1.45	0.65
APACHE II (1-point increments)	0.99	0.96 - 1.03	0.62
Baseline GCS ≤ 8	0.59	0.35 - 1.01	0.06
Renal insufficiency	1.04	0.55 - 1.95	0.90
End-stage liver disease	0.00	0	0.97
Postintubation benzodiazepines administration	0.91	0.45 - 1.86	0.80
Postintubation intermittent NMBA administration	0.35	0.08 - 1.46	0.15

ED - emergency department; BMI - body mass index; APACHE II - Acute Physiologic Assessment and Chronic Health Evaluation II; GCS - Glasgow Coma Scale; NMBA - neuromuscular blocking agents.

This is an open access article under the CC BY license (https://creativecommons.org/licenses/by/4.0/).

Table 3S - Multivariable cox regression analysis for patient location as a predictor for achieving light sedation at 24 hours; during the COVID-19 pandemic

Variables	Hazard ratio	95% confidence interval	p value
Location of intubation (ED)	0.83	0.41 - 1.69	0.61
Age (1-year increment)	1.00	0.98 - 1.02	0.81
Obesity (BMI ≥ 30kg/m^2)	1.67	0.83 - 3.36	0.15
APACHE II (1-point increments)	0.97	0.93 - 1.01	0.15
Baseline GCS ≤ 8	0.72	0.37 - 1.38	0.32
Renal insufficiency	0.41	0.16 - 1.08	0.07
End-stage liver disease	1.64	0.21 - 12.89	0.64
Postintubation benzodiazepines administration	2.23	1.09 - 4.57	0.03
Postintubation intermittent NMBA administration	1.53	0.52 - 4.47	0.44

ED - emergency department; BMI - body mass index; APACHE II - Acute Physiologic Assessment and Chronic Health Evaluation II; GCS - Glasgow Coma Scale; NMBA - neuromuscular blocking agents.

Table 4S - Multivariable cox regression analysis for patient location as a predictor for achieving light sedation at 24 hours: subgroup analysis for patients with baseline Glasgow Coma Scale > 8

Variables	Hazard ratio	95% confidence interval	p value
Location of intubation (ED)	0.61	0.31 - 1.17	0.14
Age (1-year increment)	1.00	0.99 - 1.01	0.86
Obesity (BMI ≥ 30kg/m^2)	1.02	0.66 - 1.58	0.93
APACHE II (1-point increments)	0.99	0.96 - 1.02	0.51
Renal insufficiency	0.68	0.35 - 1.31	0.25
End-stage liver disease	0.37	0.05 - 2.86	0.34
Postintubation benzodiazepines administration	1.17	0.64 - 2.15	0.61
Postintubation intermittent NMBA administration	0.71	0.25 - 1.99	0.51

ED - emergency department; BMI - body mass index; APACHE II - Acute Physiologic Assessment and Chronic Health Evaluation II; GCS - Glasgow Coma Scale; NMBA - neuromuscular blocking agents.

Table 5S - Comparison of the Sedation Agitation Scale between the emergency department and intensive care unit group at 6, 12, 24 and 48 hours after intubation

Timing after intubation	Emergency department			Intensive care unit			p value
	N total	SAS 1 - 2	SAS ≥ 3	N total	SAS 1 - 2	SAS ≥ 3	
6 hours	95	69 (72.6)	26 (27.4)	154	103 (66.9)	51 (33.1)	0.34
12 hours	93	56 (60.2)	37 (39.8)	162	75 (46.3)	87 (53.7)	0.03
24 hours	85	35 (41.2)	50 (58.8)	157	47 (29.9)	110 (70.1)	0.08
48 hours	68	18 (26.5)	50 (73.5)	146	19 (13.0)	127 (87.0)	0.02

N total - total number of patients at that time point; SAS - Sedation Agitation Scale. P value represents the comparison of Sedation Agitation Scale between the emergency department and the intensive care unit group. Results expressed as n (%).

Table 6S - Intubation technique (rapid sequence intubation *versus* non- apid sequence intubation by location and the COVID-19 pandemic

Location	Technique	Prior to COVID n (%)	During COVID n (%)	p value
ED	RSI	42 (75.0)	36 (92.3)	0.03
	Non-RSI	14 (25.0)	3 (7.7)	
ICU	RSI	18 (17.3)	33 (50.8)	< 0.001
	Non-RSI	86 (82.7)	32 (49.2)	
Total	RSI	60 (37.5)	69 (66.3)	< 0.001
	Non-RSI	100 (62.5)	35 (33.7)	

ED - emergency department; RSI - rapid sequence induction; ICU - intensive care unit. Prior to COVID-19 pandemic was defined as January 2018 to January 2020; and during the COVID-19 pandemic was defined as February 2020 to February 2022.

Table 7.1S - Sedative, analgesic, and paralytic medications used for intubation and post-intubation in the emergency department prior to and during COVID-19 pandemic.

ED (N = 95)	Prior to COVID-19 pandemic n = 56	During the COVID-19 pandemic n = 39	p value
Induction drug administration			
Xylocaine topical	3 (5.4)	0	0.14
Fentanyl	7 (12.5)	2 (5.1)	0.23
Dosage (mcg/kg)	1.1 (0.6 - 1.8)	1.0 (0.6 - 1.4)	1.00
Ketamine	30 (53.6)	28 (71.8)	0.07
Dosage (mg/kg)	1.1 (0.8 - 1.4)	1.2 (0.9 - 1.4)	0.66
Propofol	21 (37.5)	10 (25.6)	0.23
Dosage (mg/kg)	1.1 (0.9 - 1.6)	1.4 (0.9 - 1.7)	0.44
Midazolam	5 (8.9)	1 (2.6)	0.21
Dosage (mg/kg)	0.06 (0.03 - 0.1)	0.08 (0.08 - 0.08)	0.77
Rocuronium	14 (25.0)	30 (76.9)	< 0.001
Dosage (mg/kg)	0.9 (0.6 - 1.0)	1.3 (1.2 - 1.6)	< 0.001
Succinylcholine	29 (51.8)	7 (17.9)	< 0.001
Dosage (mg/kg)	1.4 (1.1 - 1.8)	1.9 (1.2 - 2.3)	0.12
Post-intubation drug administration			
Opioids	14 (25.0)	10 (25.6)	0.94
Opioid dosage (fentanyl equivalent)			
Bolus (mcg/kg)	0.9 (0.6 - 1.3)	0.7 (0.3 - 1.4)	0.81
Infusion (mcg/kg/hour)	0.8 (0.6 - 1.1)	1.3 (0.7 - 3.3)	0.29
Ketamine	5 (8.9)	11 (28.2)	0.01
Ketamine dosage			
Bolus (mg/kg)	0.4 (0.2 - 0.7)	0.1 (0.02 - 0.2)	0.03
Infusion (mg/kg/hour)	0.4 (0.3 - 0.5)	0.5 (0.5 - 0.5)	0.26
Propofol	40 (71.4)	8 (20.5)	< 0.001
Propofol dosage			
Bolus (mg/kg)	1.1 (0.3 - 1.1)	0.5 (0.3 - 0.6)	0.44
Infusion (mcg/kg/min)	25 (16 - 50)	40 (25 - 50)	0.38

Continue...

...continuation

Benzodiazepines	20 (35.7)	12 (30.8)	0.61
Benzodiazepine dosage (midazolam equivalent)			
Bolus (mg/kg)	0.04 (0.03 - 0.09)	0	-
Infusion (mg/kg/hour)	0.05 (0.04 - 0.06)	0.04 (0.03 - 0.08)	0.56
Intermittent NMBA administration	0	4 (10.3)	0.01
Method of opioid administration, peri-intubation			0.92
Bolus	8 (14.3)	4 (10.3)	
Infusion	6 (10.7)	5 (12.8)	
Both	2 (3.6)	1 (2.6)	
None	40 (71.4)	29 (74.4)	
Method of sedative administration, peri-intubation			0.81
Bolus	7 (12.5)	5 (12.8)	
Infusion	1 (1.8)	2 (5.1)	
Both	47 (83.9)	31 (79.5)	
None	1 (1.8)	1 (2.6)	

ED - emergency department; NMBA - neuromuscular blocking agent. Fentanyl equivalent parenteral dose; fentanyl 0.1mg = morphine 10mg = hydromorphone 1.5mg; Midazolam equivalent parenteral dose; midazolam 1.5mg = diazepam 5mg. Data are shown as n (%) or median (interquartile range).

Table 7.2S - Sedative, analgesic, and paralytic medications used for intubation and post-intubation in the intensive care unit prior to and during COVID-19 pandemic

ICU (N = 169)	Prior to COVID-19 pandemic n = 104	During the COVID-19 pandemic n = 65	p value
Induction drug administration			
Xylocaine topical	19 (18.3)	6 (9.2)	0.11
Fentanyl	76 (73.1)	39 (60.0)	0.08
Dosage (mg/kg)	0.8 (0.6 - 1.5)	1.0 (0.7 - 1.3)	0.37
Ketamine	36 (34.6)	32 (49.2)	0.06
Dosage (mg/kg)	0.7 (0.5 - 1.0)	1.0 (0.6 - 1.2)	0.04
Propofol	58 (55.8)	27 (41.5)	0.07
Dosage (mg/kg)	0.7 (0.4 - 1.0)	0.6 (0.4 - 0.9)	0.44
Midazolam	46 (44.2)	20 (30.8)	0.08
Dosage (mg/kg)	0.02 (0.02 - 0.03)	0.03 (0.02 - 0.04)	0.34
Rocuronium	27 (26.0)	37 (56.9)	< 0.001
Dosage (mg/kg)	0.7 (0.6 - 0.8)	1.1 (0.7 - 1.4)	< 0.001
Succinylcholine	0	0	1.00
Post-intubation drug administration			
Opioids	70 (67.3)	46 (70.8)	0.64
Opioids dosage (fentanyl equivalent)			
Bolus (mcg/kg)	0.5 (0.3 - 0.9)	0.8 (0.5 - 1.4)	0.27
Infusion (mcg/kg/hour)	0.8 (0.6 - 1.2)	0.9 (0.7 - 1.6)	0.23

Continue...

...continuation

Ketamine	3 (2.9)	5 (7.7)	0.15
Ketamine dosage			
Bolus (mg/kg)	0.6 (0.5 - 0.7)	1,2 (1.2 - 1.2)	0.22
Infusion (mg/kg/hour)	0.03 (0.03 - 0.03)	0.1 (0.06 - 0.3)	0.16
Propofol	58 (55.8)	52 (80.0)	0.001
Propofol dosage			
Bolus (mg/kg)	0.8 (0.1 - 1.5)	0.7 (0.4 - 0.8)	0.51
Infusion (mcg/kg/min)	20 (15 - 35)	30 (25 - 40)	0.003
Benzodiazepines	5 (4.8)	9 (13.8)	0.04
Benzodiazepine dosage (midazolam equivalent)			
Bolus (mg/kg)	0.04 (0.02 - 0.07)	0.04 (0.02 - 0.05)	0.64
Infusion (mg/kg/hour)	0.04 (0.01 - 0.1)	0.04 (0.04 - 0.05)	0.71
Intermittent NMBA administration	4 (3.8)	5 (7.7)	0.28
Method of opioid administration, peri-intubation			0.92
Bolus	8 (14.3)	4 (10.3)	
Infusion	6 (10.7)	5 (12.8)	
Both	2 (3.6)	1 (2.6)	
None	40 (71.4)	29 (74.4)	
Method of sedative administration, peri-intubation			0.003
Bolus	28 (26.9)	7 (10.8)	
Infusion	6 (5.8)	3 (4.6)	
Both	62 (59.6)	55 (84.6)	
None	8 (7.7)	0	

ICU - intensive care unit; NMBA - neuromuscular blocking agent. Fentanyl equivalent parenteral dose; fentanyl 0.1mg = morphine 10mg = hydromorphone 1.5mg. Midazolam equivalent parenteral dose; midazolam 1.5mg = diazepam 5mg. Data are shown as n (%) or median (interquartile range).

Table 8S - Discharge disposition of patients classified by level of sedation at 6, 12, 24 and 48 hours after intubation

	Deeply sedated	Lightly sedated	P value
6 hours after intubation, n (%)	n = 172	n = 77	
Home	50 (29.1)	28 (36.4)	0.03
Death	57 (33.1)	23 (29.9)	
Rehabilitation and long-term care institution	36 (20.9)	6 (7.8)	
Acute care hospital and institution	29 (16.9)	20 (26.0)	
12 hours after intubation, n (%)	n = 131	n = 124	
Home	33 (25.2)	45 (36.3)	0.004
Death	44 (33.6)	39 (31.5)	
Rehabilitation and long-term care institution	32 (24.4)	11 (8.9)	
Acute care hospital and institution	22 (16.8)	29 (23.4)	
24 hours after intubation, n (%)	n = 82	n = 160	
Home	16 (19.5)	55 (34.4)	0.06

Continue...

...continuation

Death	32 (39.0)	48 (30.0)	
Rehabilitation and long-term care institution	18 (22.0)	23 (14.4)	
Acute care hospital and institution	16 (19.5)	34 (21.3)	
48 hours after intubation, n (%)	n = 37	n = 177	
Home	7 (18.9)	52 (29.4)	0.33
Death	17 (45.9)	56 (31.6)	
Rehabilitation and long-term care institution	7 (18.9)	32 (18.1)	
Acute care hospital and institution	6 (16.2)	37 (20.9)	

Deeply sedated is defined as Sedation Agitation Scale 1 - 2; Lightly sedated is defined as Sedation Agitation Scale ≥ 3. Data are shown as n (%).

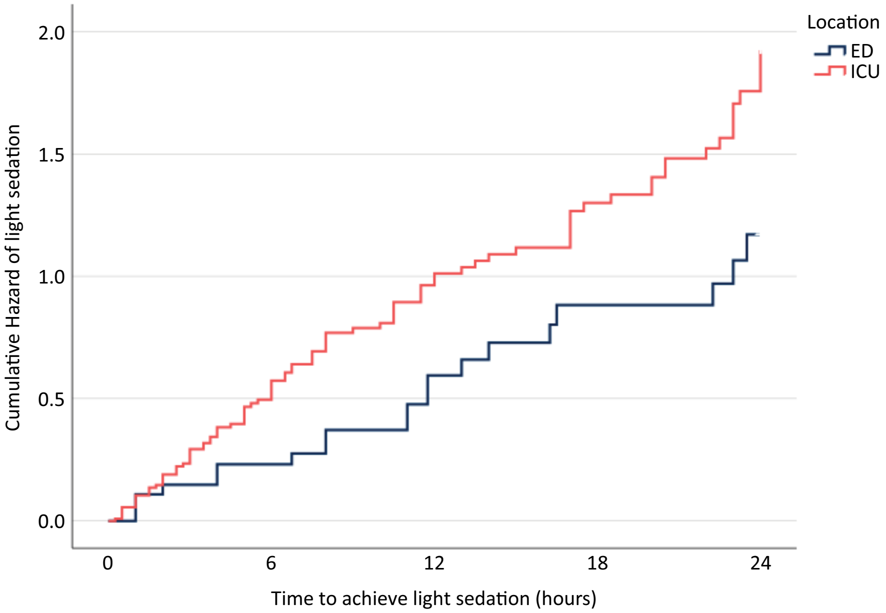

Figure 1S - Cumulative hazard of the time to achieve light sedation at 24 hours, by patients' location of intubation: Subgroup analysis for patients with baseline Glasgow Coma Scale > 8.

Within the first 24 hours after intubation, the median time to achieve light sedation was 14 hours for patients intubated in the emergency department and 7.5 hours for those intubated in the intensive care unit. The mean hazard ratio for achieving light sedation at 24 hours was 0.60 for the emergency department ($p = 0.04$; 95% confidence interval, 0.37 - 0.98). ED - emergency department; ICU - intensive care unit.

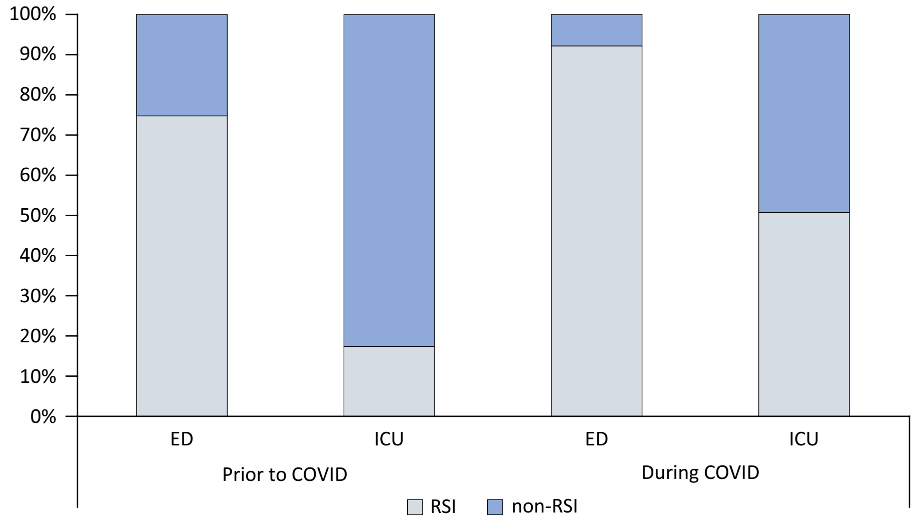

Figure 2S - Intubation technique (rapid sequence induction *versus* non-rapid sequence induction) by location and COVID-19 pandemic.

ED - emergency department; ICU - intensive care unit; RSI - rapid sequence induction. Rapid sequence induction was more frequently used in the emergency department compared to the intensive care unit. During the COVID-19 pandemic, the use of rapid sequence induction increased significantly from 75% to 92.3% ($p = 0.03$) in the emergency department, and from 17.3% to 50.8% in the intensive care unit ($p < 0.001$).
